# Supplementary material for: Characterizing cell-type spatial relationships across length scales in spatially resolved omics data
Source: Nat Commun. 2025 Jan 3;16:350. doi: 10.1038/s41467-024-55700-1 (PMC11699133; doi:10.1038/s41467-024-55700-1)
Supplement: Supplementary file 3 — Supplementary Software 1 [file 41467_2024_55700_MOESM3_ESM.zip › CRAWDAD/plots/slideseq/2_slideseq_all_trends_heatmap.pdf]

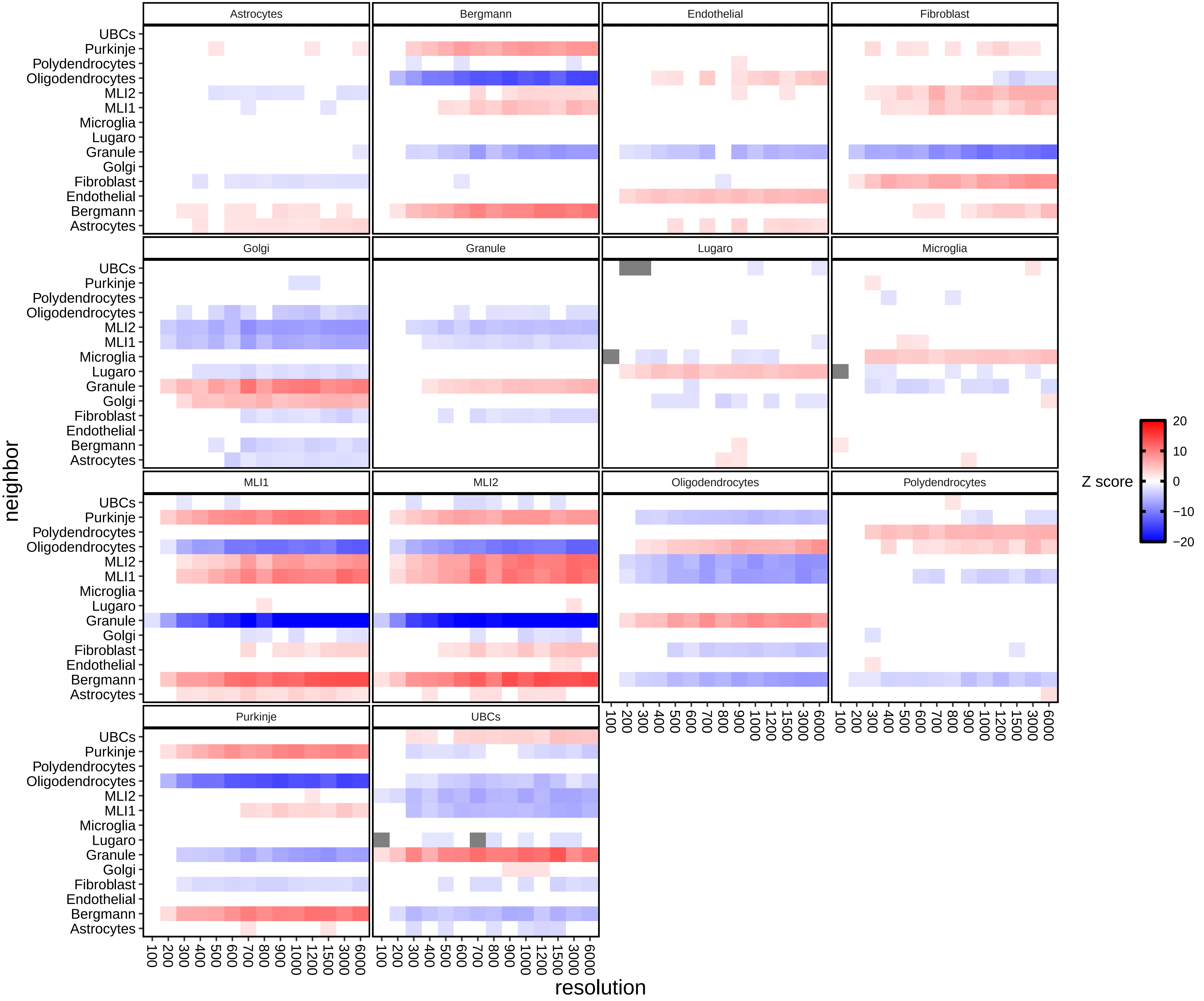
Astrocytes
Bergmann
Endothelial
Fibroblast
Golgi
Granule
Lugaro
Microglia
MLI1
MLI2
Oligodendrocytes
Polydendrocytes
Purkinje
UBCs
UBCs
Purkinje
Polydendrocytes
Oligodendrocytes
MLI2
MLI1
Microglia
Lugaro
Granule
Golgi
Fibroblast
Endothelial
Bergmann
Astrocytes
neighbor
resolution
100
200
300
400
500
600
700
800
900
1000
1200
1500
3000
6000
Z score
20
10
0
−10
−20
